# In Vivo Absorption and Lymphatic Bioavailability of Docosahexaenoic Acid from Microalgal Oil According to Its Physical and Chemical Form of Vectorization

**DOI:** 10.3390/nu16071014

**Published:** 2024-03-30

**Authors:** Leslie Couëdelo, Stephanie Lennon, Hélène Abrous, Ikram Chamekh, Corentin Bouju, Hugues Griffon, Carole Vaysse, Lionel Larvol, Gildas Breton

**Affiliations:** 1 ITERG, Nutrition Life Sciences, 33610 Bordeaux, France; h.abrous@iterg.com (H.A.); i.chamekh@iterg.com (I.C.); c.bouju@iterg.com (C.B.); h.griffon@iterg.com (H.G.); c.vaysse@iterg.com (C.V.); 2 POLARIS, 29000 Quimper, France; lionel.larvol@polaris.fr

**Keywords:** fatty acid vectorization, lymph, enterocyte, lipids, microalgal oil, long-chain omega-3, bioavailability, PUFA absorption, chylomicrons

## Abstract

Docosahexaenoic acid (DHA) is an essential fatty acid (FA) with proven pro-health effects, but improving its bioavailability is becoming a public health issue. The bioavailability of DHA from microalgal (A) oil has been comprehensively assessed, particularly in terms of the molecular structuring capabilities offered by A-oil. Here, we explored the impact of five DHA-rich formulas differing in terms of (i) molecular structure, i.e., ethyl ester (EE), monoglyceride (MG), or triglyceride (TG), and (ii) supramolecular form, i.e., emulsified TG or TG + phospholipids (PL blend) on the lymphatic kinetics of DHA absorption and the lipid characteristics of the resulting lipoproteins. We demonstrated in rats that the conventional A-DHA TG structure afforded more effective DHA absorption than the EE structure (+23%). Furthermore, the A-DHA MG and A-DHA emulsions were the better DHA vectors (AUC: 89% and +42%, respectively) due to improved lipolysis. The A-DHA MG and A-DHA emulsion presented the richest DHA content in TG (+40%) and PL (+50%) of lymphatic chylomicrons, which could affect the metabolic fate of DHA. We concluded that structuring A-DHA in TG or EE form would better serve for tissue and hepatic metabolism whereas A-DHA in MG and emulsion form could better target nerve tissues.

## 1. Introduction

Long-chain omega-3 fatty acids (*n*-3 LC PUFAs), especially eicosapentaenoic acid (C20:5n-3, EPA) and docosahexaenoic acid (C22:6n-3, DHA), have recognized health benefits. Strong evidence shows that DHA reduces the risk of developing several chronic pathologies, including cardiovascular diseases, stroke, neurodegenerative diseases, and inflammation [1,2,3,4,5,6]. DHA biosynthesis is limited in humans and animals [7,8,9], and so it has to be sourced via diet. The main dietary sources of DHA are seafood and fatty fish derivatives. However, dietary consumption data suggest that EPA and DHA intakes are less than half of the current recommendations [10,11,12]. To address the challenge of insufficient n-3 LC PUFA intake in a context where halieutic resources are declining, the food industry needs to innovate and expand its scientific research. 

Microalgal oils are a primary source of n-3 LC-PUFA oils that present a promising and sustainable alternative to fish oils [13]. Unlike fish oils, they are allergen-free and contaminant-free [14,15]. For some years now, research on increasing n-3 LC PUFA intake has turned to focus on the benefits of improving n-3 LC PUFA bioavailability by modifying the physical and chemical parameters of formulated fatty acids (FAs) in order to improve the nutritional status of the population. FA bioavailability is conditioned both by the molecular form in which the FAs are embedded and the supramolecular structure in which they are solubilized [16,17,18,19,20,21]. 

In regular diet, FAs, including DHA, are mainly provided as triglycerides (TGs) (95% of dietary lipids). However, other structures such as diglycerides (DGs), monoglycerides (MGs), ethyl esters (EEs), phospholipids (PLs), and free fatty acids (FFAs) also provide FAs. Several studies have highlighted the impact of molecular structure on FA absorption and bioavailability, but data on DHA are fragmented and no coherent consensus has emerged. Most studies agree that the EE form appears to be the least bioavailable form compared to the conventional TG structure [22,23]. However, some studies find that the hydrolysate forms, such as free fatty acids (FFAs), MGs, or diglycerides (DGs), are good candidates for increasing the DHA content in plasma but also DHA accretion in target tissues [22,24,25,26,27,28,29]. Overall, the various short- and long-term studies based on human and animal models point to a better bioavailability of DHA when it is incorporated into FFA or MG structures compared with a TG structure and even more compared with the EE form. The greater bioaccessibility observed in these studies is mostly explained by the digestion step, which is crucial for FA absorption. Dietary lipids, which are mainly TGs and to a lesser extent PLs, need to be hydrolyzed by the enzymatic action of digestive lipases in order to become absorbable [30,31]. The speed and extent of lipolysis depends on the chemical form in which FAs are integrated, i.e., TGs, PLs, FFAs, or EEs, and on the FA profile of the lipid structures. It was reported that EEs were hydrolyzed slower and less than a DHA-TG structure [32,33,34], whereas FFAs and MGs would be hydrolyzed faster and more efficiently than TGs as they are products of lipolysis that mostly bypass the digestion step [34,35]. The lipolysis level directly governs the micellization process that structures FAs into a form that is absorbable by the intestinal barrier [36].

Interestingly, a study comparing TGs vs. PLs for n-3 vectorization found that the molecular form had no influence on net intestinal absorption of FAs but did influence their incorporation into lymph [16,37] and plasma lipids, altering the size and composition of lipoproteins [18,23,38]. As lipoproteins are the carriers of fatty acids in organisms, any change in their composition, in terms of both quantity and quality, would have an impact on the metabolic fate of FAs. Thus, by structuring essential FAs, we can hope to drive their distribution to specific target tissues. In particular, the MG and PL forms, and more specifically the lyso-PC DHA, appeared to improve PUFA status in the brain and retina [26,39,40,41].

In addition to the molecular structure, the supramolecular form has also been reported to impact FA bioavailability, particularly with regards to n-3 PUFAs [19,20,36,42,43,44,45,46,47,48]. Lipid structuring has been widely used to improve the bioavailability of FAs by physically arranging food lipids in emulsions with vegetable PLs, used either as lipid transporters for essential FAs or as surfactants in lipid emulsions. The supramolecular form in which lipids are solubilized plays a role in FA absorption, which ultimately determines the bioavailability of n-3 PUFAs in lymph [19,20,36,45] and in plasma compartments [37,47]. The explanatory mechanism for this is an improvement in gastrointestinal lipolysis. Indeed, by creating lipid droplets in suspension, the emulsification process provides a large lipid–water interface available for the adsorption of pancreatic lipase and promotes faster and more efficient lipolysis compared to the bulk phase oil [36,47,49,50]. Vegetable PLs (lecithins) are good candidates for improving the interface and hence the digestibility of lipid matrices [37,47,51,52,53]. The rate of lipolysis is thus strongly influenced by the supramolecular structures of the lipid matrix [18,54,55], making gastrointestinal lipolysis a crucial step for acting on FA bioavailability. 

The bioavailability of an FA is shaped by the various upstream steps from digestion and micellization of the FA down to intestinal absorption. Studies show that the digestion step is crucial to FA absorption and dictates subsequent FA bioavailability. Lipid formulation via emulsification with lecithin or by structuring FAs in different chemical forms offers routes to improving their bioavailability. However, this link has not yet been firmly established, as there are many divergences between previous studies and, importantly, a lack of studies covering all these formulas. Moreover, most of the data available come from research focused on fish oil, and few studies have investigated the bioavailability of DHA from microalgal oils. In this context of insufficient omega-3 intake and limited halieutic resources, microalgal oils could be a useful option to vectorize DHA by different carrying structures, but the bioavailability of these forms remains to be explored. Innovative forms of DHA derived from microalgal oils could enhance DHA absorption and potentially create routes to target specific tissues. Lymph is a compartment of choice to study FA absorption and bioavailability, since after digestion, lipids are directly absorbed through the enterocytes to enter the lymphatic pathway before being metabolized by the liver. 

In this context, we assessed the intestinal absorption of DHA-microalgal oil by focusing on two factors: the molecular structure and the supramolecular form of DHA delivery. Here, the potential nutritional effect of algal oil was assessed in the lymph of rats fed a microalgal DHA-rich oil (A-DHA) in which DHA was provided either in TG form alone (A-DHA TG), blended with vegetable PLs (A-DHA PL blend), or TGs in emulsion (A-DHA emulsion), or supplied either in a monoacylglycerol structure (A-DHA MG) or in ethyl esters (A-DHA EE). Lymph was collected sequentially over 6 h post-feeding, and lymph samples were characterized by FA composition and quantification. In order to track DHA incorporation into lymph lipoproteins as the in vivo vectorization structure, we measured the incorporation of DHA in the main lipid fractions of lymphatic chylomicrons (CM), i.e., the TG and PL fractions. 

## 2. Materials and Methods

### 2.1. Materials

Omegavie^®^ DHA 800 Algae oil was sourced from Polaris (Quimper, France) and the oil emulsion (A-DHA emulsion) was produced by Polaris under patent WO 2021224940A1. Polaris synthesized the A-DHA MG, A-DHA EE, and A-DHA PL blend formulas. The lipid characterization, as glyceridic composition, fatty acid profile, and oil structure, was determined according to the standardization methods of standard IUPAC 6.002 [56] and French standards NF EN ISO 14105 [57] and NF EN ISO 12966 [58,59], respectively (Table 1).

Acetyl chloride, acetic acid, potassium chloride (KCl), sodium carbonate (Na_2_CO_3_), sodium chloride (NaCl), sodium methoxide (CH_3_ONa), sulfuric acid (H_2_SO_4_), and all organic solvents used, i.e., di-ethyl ether, heptane, hexane, iso-octane, and methanol (analytical or HPLC grades) were provided by Thermo Fisher Scientific (Strasbourg, France). Internal standard 1,2-diheptadecanoyl-sn-glycero-3-phosphatidylcholine (PC 17:0) and 1,2,3-triheptadecanoyl-sn-glycerol (TG 17:0) were obtained from Avanti Polar Lipids Inc. (Alabaster, AL, USA). 

Ketamine, xylazine, buprenorphine, and sodium pentobarbital were provided by Axience (Pantin, France).

### 2.2. Lipid Formulations

Five formulations were obtained from a DHA-rich microalgal oil, i.e., the algal (A) re-esterified triglycerides (A-DHA TG) or emulsion (A-DHA emulsion), or DHA vectorized by a different lipid structure, i.e., algal ethyl ester (A-DHA EE), a blend of algal phospholipids (A-DHA PL blend), or algal monoglyceride (A-DHA MG). See Table 1 for composition data.

### 2.3. Experimental Design—Animal and Surgical Procedures

The experiments and procedures were approved by the governing French Ministry under approval No. DAP38317-V5-2022083015242376 recorded by APAFiS, and were carried out in compliance with the local Ethics Committee of the University of Bordeaux (France) (CEEA50). The number of animals required for the study was decided with input from the Institutional Review Board on setting up the research project. The number of animals was obtained on the basis of previous studies carried out on lymph absorption and within the framework of the 5R rules, which allowed us to increment 6 rats per group. Male Wistar rats (8 weeks old, body weight 368.2 ± 16.7 g) were obtained from Janvier (Saint-Berthevin, France) and were randomly assigned to one of the 5 dietary groups (A-DHA TG, A-DHA EE, A-DHA PL blend, A-DHA emulsion, A-DHA MG). Animals were treated in accordance with the European Communities Council Guidelines for the Care and Use of Laboratory Animals (2010/63/EU). All experiments complied with the Guidelines for the Handling and Training of Laboratory Animals. Before starting the experiment, the rats were housed for at least 3 days in a constant temperature and humidity-controlled environment and with free access to food and water. The day before surgery, rats were fed a fat-free diet (Epinay, France) and had free access to water. On the day of surgery, each rat was placed under anesthesia by an injection of ketamine/xylazine (100/10 mg/kg each). After visualization of the main mesenteric lymph duct, a polyethylene catheter (0.95 mm × 15 cm; Biotrol, Paris, France) was inserted, as described in [47,60]. After surgery, rats were placed in individual restraining cages in a warm environment with water freely available. To prevent pain, the rats received an intra-peritoneal injection of buprenorphine (0.02 mg/kg) 1 h before and 2 h after surgery. 

In each experiment, an equivalent amount of 300 mg of DHA was administered to each rat (n = 6 rats per group), whatever the DHA-rich microalgal oil formulation used. Lymph was collected hourly for 6 h post-feeding. At the end of the experiment, rats were euthanized by an intra-peritoneal injection of sodium pentobarbital.

The sequential collection of lymph specimens served to define the kinetics of intestinal absorption of n-3 according to DHA-rich microalgal oil formulation administered, as well as the maximum lymphatic concentration (Cmax) of n-3 PUFAs and the time at which this maximal n-3 PUFA concentration was reached (Tmax).

### 2.4. Fatty Acid Profile and n-3 LC PUFAs Composition of Lymph Fatty Acids

Total fatty acid composition of lymph collected at different times during the 6 h post-feeding period was directly obtained by the method described in Lepage and Roy [61]. 

The resulting FA methyl esters (FAMEs) were analyzed by GC (TRACE GC; Thermo Scientific, Waltham, MA, USA) equipped with a flame ionization detector (FID) and a split injector. A fused-silica capillary column (BPX 70, 60m × 0.25mm i.d., 0.25 μm film; SGE, Illkirch, France) was used with hydrogen as the carrier gas (inlet pressure: 120 kPa). The split ratio was 1:33. The column temperature program was as follows: from 150 °C, the temperature was ramped to 200 °C at 1.5 °C/min, then held for 30 min before being ramped to 20 °C/min up to 225 °C and held for 15 min. The injector and detector were both held at 250 °C. GC peaks were integrated using Chromeleon software version 7.2 (Thermofinnigan, Courtaboeuf, France). FAs were quantified using tri-heptadecanoic acid as an internal standard, and added at 10% of the lipid weight before the (trans)methylation procedure.

### 2.5. DHA Incorporation in TG and PL Fractions of Lymph

The collection of lymph samples obtained during the kinetics of lipid uptake (6 points per animal) were pooled for each animal. The resulting single sample per animal represents the DHA absorption over the 6 h post-feeding period. TGs and PLs are the major lipid fractions in lymph and therefore the major constituents of lymph chylomicrons, representing around 85% and 10% of lymph lipids, respectively. 

Total lipids were first extracted using the Folch method [62]. Briefly, the extraction was performed in chloroform/methanol (2/1, *v*/*v*) under stirring, at room temperature. After 1 h, 0.2 volume of KCl (0.8% in water, *w*/*v*) per volume of the extraction mixture was added, and the samples were centrifuged (1050× *g*, 5 min, 4 °C) to separate the chloroform and hydroalcoholic phases. The aqueous phase was removed and the chloroform phase containing the total lipids was filtered with chloroform/methanol (2/1, *v*/*v*), and evaporated under vacuum with a rotary evaporator. The extracts were then re-dissolved in chloroform, filtered, and then dried under nitrogen. The extracted lipids were dissolved in chloroform/methanol (2/1, *v*/*v*) and stored at −20 °C until analysis.

The lymph PL, FFA, TG, and cholesterol ester (CE) fractions were separated by thin-layer chromatography (20 × 20 cm glass plates pre-coated with 60H silica gel(Merck KGaA, Darmstadt, Germany)) using a solvent mixture composed of hexane/diethyl ether/acetic acid (80/20/1, *v*/*v*/*v*). After vaporization of 2,7-dichlorofluorescein and visualization under UV light, spots corresponding to PLs, FFAs, TGs, and CE were identified by external standards spotted on the plate and extracted from the silica gel by addition of 2·5 mL chloroform/methanol (2/1, *v*/*v*). After homogenization and centrifuging the different lipid fractions (1050× *g*, 5 min, 20 °C), the organic phase was collected. Then, 100 μL distilled water and chloroform/methanol (2/1, *v*/*v*, 2 mL) were added to the silica gel phase. The extraction step was repeated, and the organic phase was collected. Lipid extraction from silica gel ended by adding 2 mL of methanol to the silica gel phase, followed by homogenization and centrifugation (1050× *g*, 5 min, 20 °C). The organic phases were then collected and dried under N. Finally, 2,7-dichlorofluorescein was removed using 0·4 mL of a KCl solution (0.8% in distilled water *w*/*v*) and 2 mL of chloroform/methanol (2/1, *v*/*v*). The organic phase was washed twice by adding 0·8 mL of a mixture composed of chloroform/methanol/KCL 0.8% in distilled water (15/240/235, *v*/*v*/*v*). Samples were stored at −20 °C until analysis.

The fatty acid composition of the triglyceride (TG) and phospholipid (PL) fractions was determined by gas chromatography (GC-FID) after trans-methylation using the Castro-Gomez method [63], and quantified using internal standards, i.e., TG C17:0 to determine the TG fraction and PL 17:0 to determine the PL fraction. 

Data are reported as DHA concentration (µg/µL lymph) and DHA proportion (%) in total FAs in the lipid fraction for the 5 formulas studied, i.e., A-DHA TG, A-DHA EE, A-DHA PL blend, A-DHA emulsion, A-DHA MG).

### 2.6. Statistical Analysis 

Data were expressed as means with standard deviations (SD) and were analyzed using XLStat software version 2023.2.0 to evaluate the kinetics of absorption of DHA over the 6 h following lipid administration. Furthermore, the kinetics curve was also used to determine (i) the area under the curve (AUC; expressed in mg × h/mL), calculated according to the trapezoidal method, to assess the amount of DHA absorbed, and (ii) the maximum lymphatic concentration of DHA (Cmax) and the maximum time taken to reach it (Tmax). *p*-values lower than 0.05 were considered statistically significant.

## 3. Results

### 3.1. Influence of the Lipid Carrier on the Intestinal Absorption of DHA 

#### 3.1.1. Total Fatty Acid Absorption

**Figure 1:**
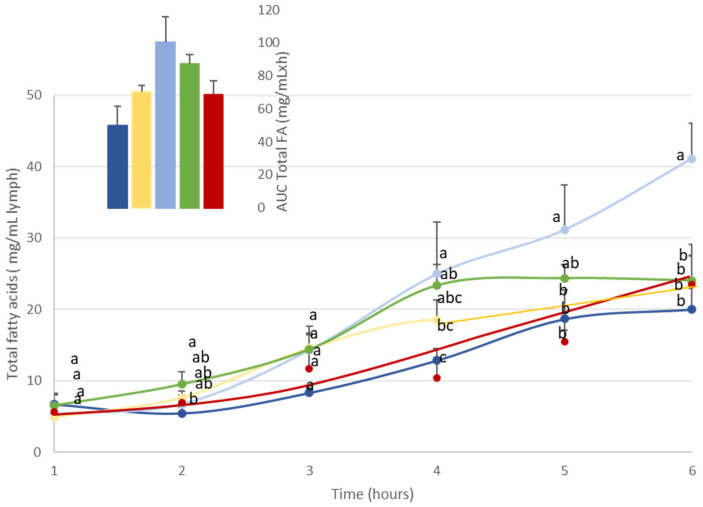
Kinetics of the intestinal absorption of lipids (mg/mL lymph) in rats subjected to a lymphatic duct fistula (n = 6 rats/group). Rats were provided a single bolus of various DHA-microalgal formulas (equivalent to 300 mg DHA/animal) where DHA was vectorized as triglyceride either in oil phase (A-DHA TG: 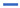), emulsified (A-DHA emulsion: 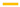), or blended to vegetable phospholipids (A-DHA PL blend: 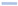), compared to DHA vectorized as ethyl ester form (A-DHA EE: 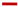) or a monoglyceride structure (A-DHA MG: 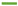). Data are presented as means ± SEM. a, b, c: values are significantly different between the 5 groups at a given timepoint (*p* < 0.05; ANOVA, Mann–Whitney post hoc test). Inset represents the area under the curve (AUC) for the 5 rat groups, which show no significant between-group difference.

The time-course kinetics of total fatty acids was determined from 1 to 6 h after DHA administration via the lipid formulations. DHA was supplied either by crude TGs, or in emulsion, or as EEs or MGs or in a vegetable oil PL blend (Figure 1).

In all groups, lipid administration induced an increase in total FA concentration from as early as 3 h post-administration. 

For most formulations, the maximal concentration of total FAs in lymph raised 20 mg/mL of lymph. The time to reach Cmax depended on the lipid structure and formulation. More precisely, when DHA was vectorized by TG structure (A-DHA TG), total lipid concentration reached a plateau at 5 h post-administration, whereas when DHA was provided in emulsion or in MG structure, the plateau was reached 1 h earlier (Tmax = 4 h). In contrast, when DHA was provided as EE, Tmax was reached 1 h later (Tmax = 6 h). 

Note that when DHA (mostly TG structure) was combined to vegetable PLs, the Cmax was doubled (Cmax = 40 mg/mL) compared to the other groups, and never reached a steady state until the end of lipid absorption (Tmax = 6 h).

Furthermore, analysis of the AUC data (insert, Figure 1) showed that AUCs varied by a factor of two depending on lipid formulation. The AUC was found to be lowest when DHA was provided as a TG structure (50 mg/mL·h), whereas it was significantly higher when DHA was provided in emulsion or EE forms (70 mg/mL·h). The AUCs relative to the overall lipid absorption were higher in the MG-vectorized DHA group (87 mg/mL·h) or when microalgal TG was combined with vegetable PLs (100 mg/mL·h).

#### 3.1.2. Lymphatic Recovery of DHA

**Figure 2:**
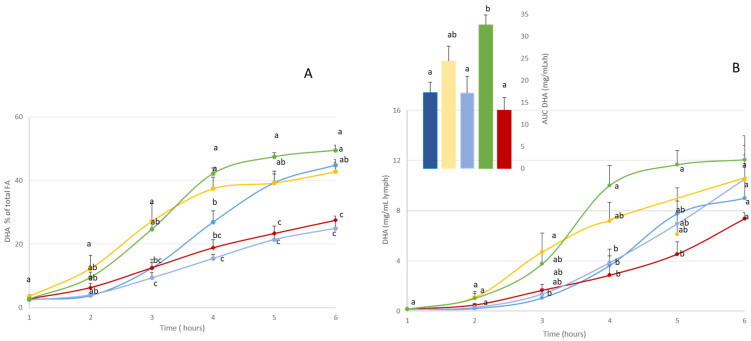
Kinetics of the intestinal absorption of DHA, i.e., (**A**) % of total fatty acids, (**B**) mg/mL lymph, in rats subjected to a lymphatic duct fistula (n = 6 rats/group). Rats were provided a single bolus of various DHA-microalgal formulas (equivalent to 300 mg DHA/animal) where DHA was vectorized as triglyceride either in oil phase (A-DHA TG: 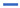), emulsified (A-DHA emulsion: 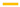), or blended to vegetable phospholipids (A-DHA PL blend: 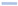), compared to DHA vectorized as an ethyl ester form (A-DHA EE: 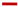) or monoglyceride structure (A-DHA MG: 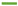). Data are presented as means ± SEM. a, b, c: values are significantly different between the 5 groups at a given timepoint (*p* < 0.05; ANOVA, Mann–Whitney post hoc test). DHA: docosahexaenoic acid. Inset represents the area under the curve (AUC) for the 5 rat groups. a, b, c: significantly between-group differences (*p* < 0.05; ANOVA, Mann–Whitney post hoc test).

To compare DHA bioavailability according to lipid structure, DHA was monitored in the lymph compartment. Figure 2 shows the kinetics of DHA absorption in the lymph compartment over the 6 h following lipid administration, both qualitatively (Figure 2A: % of total fatty acids) and quantitatively (Figure 2B: mg/mL/g lipid intake).

The time-course kinetics showed that over the 6 h following lipid administration, the lymph was progressively enriched in DHA, reaching a plateau at the end of lipid absorption (between 4 and 6 h post-administration). 

In qualitative terms (Figure 2A), DHA was gradually incorporated into lymph lipids, regardless of the formula used. At the end of the phase, DHA represented between 27 and 50% of total fatty acids, depending on the formula used. More precisely, the A-DHA PL blend and A-DHA EE groups had the lowest proportion of DHA (around 27% at T 6 h), while the A-DHA emulsion, A-DHA TG, and A-DHA MG groups presented the highest DHA proportions (43%, 45%, and 49% of total fatty acids, respectively). 

In quantitative terms (Figure 2B and insert), when the DHA levels plateaued, the Cmax values differed between the formulas used. More precisely, the A-DHA EE group showed the lowest DHA incorporation characterized by a Cmax = 7 mg/mL at T 6h, whereas A-DHA TG (9 mg DHA/mL), A-DHA PL blend (10 mg DHA/mL), and A-DHA emulsion (10 mg DHA/mL) presented intermediate Cmax values and the A-DHA MG formula shoed the highest Cmax (12 mg DHA/mL).

Note that the A-DHA MG and A-DHA emulsion showed a different kinetic DHA absorption to the other groups, particularly in terms of curve shape. The Cmax values were similar in both these groups (10 and 12 mg/mL, respectively) and tended to be higher than the Cmax values observed in the A-DHA EE and A-DHA TG groups (7 and 9 mg/mL, respectively), and the Tmax values were 1–2 h earlier than all groups (at 4 h versus 5–6 h for the other groups). 

The AUC data (Figure 2B) confirmed that “net” intestinal absorption of DHA was lowest for the A-DHA EE group (13 mg/mL·h). The A-DHA MG group had a significantly higher AUC (32 mg/mL·h) than the A-DHA emulsion (24 mg/mL·h), A-DHA TG (17 mg/mL·h), and A-DHA PL blend (17 mg/mL·h) groups. We noted a trend towards higher DHA bioaccessibility values which tended to be higher in the A-DHA emulsion and A-DHA MG groups.

#### 3.1.3. DHA Incorporation in the Main Lipid Fractions of Lymph

**Figure 3:**
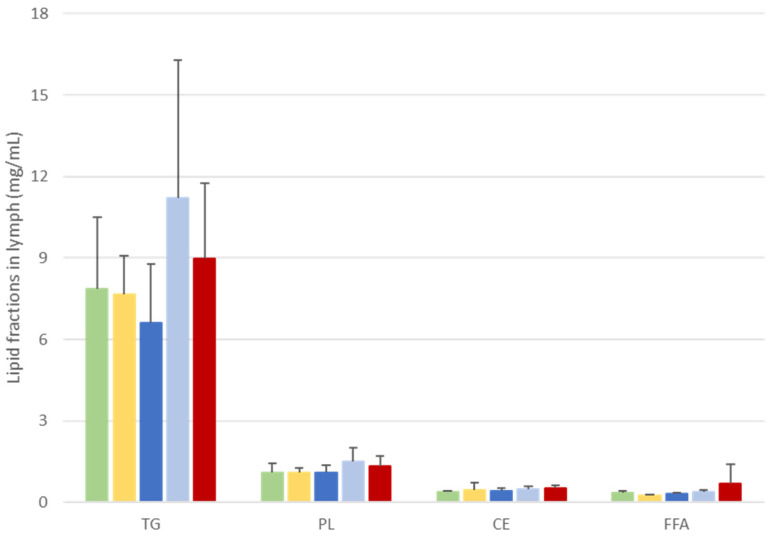
Characterization of lymph lipids in terms of lipid fractions at 6 h after lipid administration in rats with lymphatic duct fistula (n = 6 rats/group). Rats were provided a single bolus of various DHA-microalgal formulas (equivalent to 300 mg DHA/animal) where DHA was vectorized as triglyceride either in oil phase (A-DHA TG: 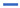), emulsified (A-DHA emulsion: 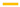), or blended to vegetable phospholipids (A-DHA PL blend: 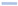), compared to DHA vectorized as ethyl ester (A-DHA EE: 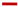) or a monoglyceride structure (A-DHA MG: 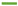). Data are presented as means ± SEM. Values were not significantly different between groups (*p* < 0.05; ANOVA, Mann–Whitney post hoc test). TG: triglycerides, PL: phospholipids, CE: cholesterol esters, FFA: free fatty acids.

Lymph is composed of TGs, PLs, and to a lesser extent cholesterol esters (CEs) and free fatty acids (FFAs). The lipid composition of the lymph was determined in terms of proportions of lipid fractions (TGs, PLs, CEs, and FFAs), as plotted in Figure 3. 

Regardless of the formula administered, the main lipid fractions in lymph were first TGs (78–82% of lymph lipids) and then, to a lesser extent, PLs, (11–13% of lymph lipids). FFAs and CEs represented less than 5% of lymph lipids.

These proportions fluctuated slightly between groups, and particularly the TG fractions. Indeed, TG concentrations tended to be higher in the A-DHA PL blend and A-DHA EE groups (11 and 9 µg/µL lymph, respectively) compared to other groups (which averaged 7.4 µg/µL).

We tracked the incorporation of DHA = in TGs and PLs, which are the major lipid fractions in the lymphatic lipoproteins, i.e., chylomicrons (Table 2).

- DHA incorporation in lymph TGs

In qualitative terms, DHA accounted for 23–45% of total FAs in the lymph TG fraction (Table 2). DHA was low in the lymph TGs of A-DHA EE and A-DHA PL blend groups, whereas it was significantly higher when DHA was vectorized in A-DHA MG, A-DHA TG, and A-DHA emulsion. In quantitative terms, DHA concentrations were similar between groups, ranging from 2.3 to 3.5 µg/µL lymph.

- DHA incorporation in lymph PLs

PLs are the second major fraction of lymph lipids. They are the structural component of chylomicrons and are located at the lipoprotein interface. We therefore also tracked DHA in the PL fraction, which along with TGs form the chylomicrons.

Table 2 shows that, qualitatively, DHA accounted for 3.4% to 10.4% of total FAs in lymph PLs. DHA was less well incorporated in the A-DHA EE and A-DHA PL blend formulas (on average 3.45% of total FAs) but significantly better incorporated in PLs from the A-DHA MG (10.4%) and A-DHA emulsion (9.9%) formulas and moderately well incorporated in PLs in the A-DHA TG group (6.2%).

Quantitatively, DHA concentrations ranged between 45 and 124 ng/µL lymph. More precisely, DHA concentrations in PLs were significantly higher in A-DHA MG (124 ng/µL lymph) and A-DHA emulsion (109 ng/µL lymph) groups compared to A-DHA EE (45 ng/µL lymph), A-DHA PL blend (55 ng/µL lymph), and A-DHA TG groups (68 ng/µL lymph).

## 4. Discussion

Microalgal oils are currently attracting growing interest as new sustainable omega-3 sources of promising alternatives to fish oils to help meet the nutritional n-3-LC PUFA requirements of the population [13,14]. Studies on the bioavailability of DHA have almost exclusively focused on fish oils, so we still have only fragmented data on microalgal oils. In this context, here, we used a rat model to determine the lymphatic absorption of DHA from microalgal oils according to its molecular and supramolecular forms of vectorization during a 6 h post-prandial time-course. We worked with Omegavie^®^ algae (A) that offer a comprehensive range of microalgal oils, from natural extracts to concentrates containing up to 800 mg of TG-DHA per gram. The microalgal-source DHA was produced in different lipid structures, i.e., ethyl ester (A-DHA EE), monoglyceride (A-DHA MG), and triglyceride (A-DHA-TG), based on internal processes and patents. In addition to the impact of the molecular structure on DHA vectorization, we also studied the impact of the supramolecular structure of the source DHA based on emulsified TG-DHA (A-DHA emulsion) and TG-DHA blended with vegetable oil PLs (A-DHA blend PL). Rats that underwent surgery with a lymphatic duct fistula were given a single bolus of each DHA formula designed to ensure that DHA intake was strictly the same in each formula and represented 300 mg/rat. The endogenous proportion of lymph DHA is negligible (less than 0.3% of total FA), and so any DHA increase in this compartment had to come from dietary intake. We are aware that the number of animals was relatively limited, at just six rats per group, which created non-negligible variability in some of our results that does not translate into more significant differences. A larger number of animals could have brought out more differences in this study, but ethical imperatives restricted the number of animals that we could use, which may be a limitation in this work.

We clearly showed that the administration of lipids in any form induced an increase in postprandial lipemia in the lymph over the 6 h following lipid formula administration. Here, considering DHA as the ‘target molecule’ for its nutritional value and pro-health effects, the use of DHA-rich formulas significantly enriched lymph in DHA. As previously reported in the literature, the fatty acid composition of lymph reflects the lipid composition of the diet ingested [64,65], and the DHA inter-group variability in DHA observed here in rat lymph (27–50%) was representative of the FA composition of the formulas ingested. We found a direct impact of molecular structure on lipid absorption. More precisely, although the AUC values were similar between groups, the kinetics of lipid appearance in lymph differed according to their form of vectorization. The Cmax value for total FAs was two-fold higher when TGs were associated with PLs (A-DHA with PL; 40 mg/mL) than for the other formulas (20 mg/mL). The analysis comparing net intestinal absorption of DHA found that the TG structure, whether blended or not with PLs (A-DHA TG and A-DHA PL blend), resulted in an intermediate DHA absorption value compared with the A-DHA MG and A-DHA emulsion groups and the A-DHA EE group. Tmax values (time to reach Cmax) were 2 h faster when the DHA was vectored in emulsion and by MG structure (Tmax = 4 h) than with the EE structure or the TGs blended with PLs (Tmax = 6 h). Our results indicate that the emulsification of lipids and the MG structure were the best formulas for improving the intestinal absorption of DHA. Our data are in line with previous work in which the supramolecular structure was found to influence the intestinal absorption and bioavailability of FAs. In particular, the emulsifying fish oil was found to enhance the absorption of n-3 LC PUFAs [19,42,46,66] and to shorten significantly the time to peak of FA absorption (Tmax) compared to the bulk-phase oil. Some authors explain the increase in bioavailability in terms of links to the digestion step, highlighting that preformed lipid droplets lead to more efficient gastric emptying [67,68]. Furthermore, the presence of a lipid/water interface promotes the anchoring of pancreatic lipase, which is necessary for TG hydrolysis and in turn promotes the micellization of FAs [30,69]. Note, however, that DHA conformation generates steric hindrance that limits its access to lipases during the digestion process [30,64,70]. As emulsification creates a larger interface, it facilitates lipid access to lipases and thus the lipolysis of DHA-containing structures. Here, the emulsification process made it easier to generate lipid droplets during the digestion step and therefore produce the larger interface that is essential to facilitate the anchoring and activity of the lipases involved in the lipolysis step [30,69,70]. By promoting the action of lipase and lipolysis, the hydrolysis products (MGs and FFAs) will be generated faster and thus more quickly micellized into mixed lipid/bile salt micelles, which is critical for promoting intestinal FA absorption.

On the other hand, the absorption of FAs and DHA was also improved by modifying the molecular structure of the lipid. Numerous studies posit that PL-DHA could be an interesting structure for the targeted vectorization of DHA [26,40,41], but here we did not examine DHA vectorization by PLs (PL-DHA type), because microalgae sources do not biosynthesize the PL structure. We designed another formula blended with vegetable oil PL; however, we cannot compare this structure against the data from the literature using PLs as a vector for DHA, but only compare its impact on the supramolecular form of DHA. As the microalgal matrix does not provide this type of structure, we did not include a PL-DHA group to serve for comparative analysis because it would be derived from marine sources (krill, for example), whereas the matrix studied here is exclusively sourced from microalgae, which again may be a limitation of this study.

This study found evidence that the MG structure appeared to better vectorize FAs than the conventional TG structure and far better vectorize FAs than the EE structure. The better absorption observed here with MG-vectorized DHA (the A-DHA MG formula) was not due to better digestibility but rather to optimal FA solubilization and faster FA absorption by intestinal cells. As MGs are lipid hydrolysates, they can mostly bypass the lipolysis step to go directly in the micellization phase and thus be absorbed faster in the gut [26,27,28]. Lipases are stereospecific for the external positions of the glycerol backbone. However, when grafted at the external position of glyceride structures, DHA generates a stereochemical hindrance that limits the hydrolysis of the ester bond. Thus, due to the presence of DHA on the external position of our formula, only a small part of the s*n*-1(3)DHA-MG formula would effectively be hydrolyzed. In this instance, the combination of faster micellization and stereochemical DHA hindrance means that the ultimate profile of our mixed micelles would mainly be composed of non-hydrolyzed s*n*-1(3)DHA-MG and a small proportion of DHA-FFA. 

At a cellular level, the stronger and faster FA uptake provided by the MGs and emulsion formulas may induce some metabolic changes during the synthesis of lymphatic lipoproteins (chylomicrons; CMs) and ultimately modify the morphological characteristics of lymphatic CMs [18,36]. As CMs are vectors of FAs from lymph to target tissues via the bloodstream, they can be analyzed in order to predict the lipid form of FA vectorization. After being absorbed by the intestinal mucosa, FFAs and MGs are re-used in the enterocytes to synthesize mostly TGs (85% of total lipids) that form the core of CMs, but also some PLs (around 10% of total lipids) that form the CM membrane [71,72]. By monitoring the incorporation of DHA into lipid fractions of lymph, we obtain an overview of the lipid characteristics of CMs in which DHA will be vectorized to tissues. 

The TG fraction tended to be higher in A-DHA PL blend (+57%) and A-DHA EE groups (+28%) than in the TG, MG, and emulsion formulas. However, the A-DHA TG, A-DHA MG, and A-DHA emulsion structures enabled to improve DHA content in lymphatic TG by 40%. Only the MG and emulsion structures enabled an improvement in DHA content in lymphatic PLs by 50%. 

The enrichment in DHA that occurred in both the TG and PL lymphatic fractions followed the first observation of improved FA uptake by enterocytes and could induce some metabolic changes related to the lipid synthesis pathways in cells. After intestinal absorption, FAs are predominantly incorporated into the ‘re-synthesized’ TGs via the main metabolic pathway of ‘2-MG’. The second more minor metabolic G3P pathway is involved in the synthesis of TGs but also PLs, and it occurs during the interprandial period or at the end of lipid absorption [73]. Thus, the presence of DHA-PL in CMs with A-DHA MG and A-DHA emulsion formulas would be the result of the activation and the earlier stimulation of this second metabolic pathway. Activation of the G3P pathway enables a threefold increase in DHA incorporation into the lymph PLs of CMs. PLs are key components of the CM membrane, and so any change in FA composition in the PL fraction will also modify the characteristics of the CM membrane and thus the metabolic fate of the FAs. These observations are in agreement with the previous work where activation of both the 2-MG and G3P pathways quickly compensated for the ‘influx’ of FAs in the enterocyte following the improved lipolysis [18,37].

The kinetics of CM synthesis have been described as a two-step process, starting first with an increase in their diameter as lipids were absorbed, particularly at the beginning of the absorption process, which is followed, beyond a certain diameter, by the stimulated synthesis of the number of CM particles. Based on the literature, we conclude that the CMs of both the TG PL blend formula and the EE form were either larger in diameter with a denser core of TGs, or more numerous compared to the CMs in the A-DHA MG and A-DHA emulsion groups. The CMs produced by the TG-DHA formula appear to be intermediate, with medium-sized CMs rich in TG-DHA. In contrast, the CMs rich in PL-DHA and TG-DHA produced with the A-DHA emulsion and A-DHA MG formulas could lead to different patterns of DHA vectorization in the organism. For instance, it has been reported that PL-DHA further promotes the DHA incorporation into nerve tissues such as the retina or brain [26,40,41,46], whereas TG-DHA could more likely be metabolized by the liver, as hypothesized by [37]. 

## 5. Conclusions

This study clearly demonstrated that the molecular structure obtained with microalgal oil (MGs, EEs, or TGs) to carry DHA but also the supramolecular structure (TG emulsified or blended with vegetable PL) modulated the digestibility of DHA-containing structures and directly influenced the absorption and metabolic fate of DHA. The DHA-TG formula was able to vectorize DHA similarly when carried alone or blended with vegetable PLs and was found to be a compromise compared to the other structures assessed (EEs, MGs, or emulsified). DHA vectorization by the EE structure (the A-DHA EE formula) was the least conducive to DHA bioavailability, whereas the A-DHA MG and A-DHA emulsion formulas were the systems best placed for carrying DHA. A-DHA MG and A-DHA emulsion formulas worked in different ways to improve the digestion and absorption steps and consequently enhance the intestinal FA absorption process resulting in more TG-DHA-dense and PL-DHA-dense lipoproteins. By modifying the characteristics of CMs, the Omegavie® DHA-TG formula, blended or not with PLs, would be better directed to the tissues and hepatic metabolism, whereas the emulsified DHA-TG and DHA-MG emulsion formulas would be more specifically directed to nerve tissues (retina or brain tissue).

## 6. Patents

The Omegavie^®^ microalgal oil emulsion was developed by Polaris under patent No. WO 2021224940A1.

## Figures and Tables

**Table 1:** Lipid characterization of microalgal DHA-rich formulas, in lipid fractions (%) and EPA/DHA composition (mg/g).

	A-DHA TG	A-DHA Emulsion	A-DHA EE	A-DHA PL Blend	A-DHA MG
Lipid fractions (%)					
TGs	67.1	69.9	0	62.6	8.5
PLs	0	0	0	21.1	0
MGs	1.4	1.2	0	1.2	73.2
DGs	29.5	26.8	0	15.1	13.9
EEs	0	1.5	100	0	0
FFAs	0	0	0	0	3.2
Others	2	0.6	0	0	1.2
FA composition (mg/g as FA)					
DHA	835	311	552	503	595
EPA	26	12	5	11.5	9
Proportion of DHA in the internal position of the lipid structure (% sn2/% sn1/3 + sn2 positions)					
In TG fraction	34.0%	34.0%	0.0%	34.0%	19.0%
In MG fraction	0.0%	0.0%	0.0%	0. 0%	4.8%
In DG fraction	50.5%	50.5%	0.0%	50.5%	39.3%

TGs: triglycerides; PLs: phospholipids; MGs: monoglycerides; DGs: diglycerides; EEs: ethyl esters; FFAs: free fatty acids. sn indicates the stereochemical position of the FA in the overall lipid structure, where sn-1(3) corresponds to the external position and sn-2 the internal position of the lipid molecule.

**Table 2:** Proportions (% in total FA) and concentrations (in µg or ng/µL lymph) of DHA in the main lipid fraction of lymph (TGs and PLs), over the first 6 h after lipid administration in rats with lymphatic duct fistula (n = 6 rats/group).

A-DHA TG	A-DHA EE	A-DHA PL Blend	A-DHA MG	A-DHA Emulsion
Mean ± SEM	Mean ± SEM	Mean ± SEM	Mean ± SEM	Mean ± SEM
% DHA in the main lipid fraction				
Lymph TGs (% of total FA)				
40.5 ^ab^ ± 1.6	25.1 ^ab^ ± 1.2	23.2 ^a^ ± 1.2	42.7 ^b^ ± 3.2	45.1 ^b^ ± 1.9
Lymph PLs (% of total FA)				
6.2 ^ab^ ± 0.4	3.4 ^a^ ± 0.4	3.5 ^a^ ± 0.3	10.4 ^b^ ± 1.9	9.9 ^b^ ± 0.9
Quantification of DHA in the main lipid fraction				
Lymph TGs (µg/µL lymph)				
2.7 ^a^ ± 0.4	2.3 ^a^ ± 0.3	2.7 ^a^ ± 0.6	3.5 ^a^ ± 0.6	3.4 ^a^ ± 0.3
Lymph PLs (ng/µL lymph)				
68.4 ^ab^ ± 10.1	45.0 ^a^ ± 7.2	55.2 ^a^ ± 11.4	124.2 ^ab^ ± 36.3	108.7 ^b^ ± 9.2

Rats were provided a single bolus of various DHA-microalgal formulas (equivalent to 300 mg DHA/animal) where DHA was vectorized as triglyceride either in oil phase (A-DHA TG), emulsified (A-DHA emulsion), or blended to vegetable phospholipids (A-DHA PL blend), compared to DHA vectorized as ethyl ester (A-DHA EE) or a monoglyceride structure (A-DHA MG). Data are presented as means ± SEM. a, b: values are significantly different between the 5 groups (*p* < 0.05; ANOVA, Mann–Whitney post hoc test). TGs: triglycerides, PLs: phospholipids.

## Data Availability

The data presented in this study are available on request from the corresponding author due to confidentiality restrictions.
